# Identifying gene expression predictive of response to neoadjuvant endocrine therapy in early breast cancer

**DOI:** 10.1007/s10549-025-07693-8

**Published:** 2025-04-09

**Authors:** Kaori Hidaka, Lisa Goto-Yamaguchi, Aiko Sueta, Mai Tomiguchi, Yutaka Yamamoto

**Affiliations:** 1 Department of Thoracic Surgery and Breast Surgery, Graduate School of Medical Sciences, Kumamoto University, Kumamoto, Japan; 2 Department of Breast and Endocrine Surgery, Kumamoto University Hospital, 1-1-1 Honjo, Chuo-Ku, Kumamoto, 860-8556 Japan; 3 Department of Breast Surgery, Wajiro Hospital, Fukuoka, Japan

**Keywords:** Breast cancer, ER-positive/HER2-negative, Gene signature, Neoadjuvant endocrine therapy, Predictive biomarker

## Abstract

**Purpose:**

Estrogen receptor (ER)-positive breast cancer is the most common subtype, accounting for approximately 80% of cases, with endocrine therapy as the standard postoperative treatment. However, despite risk-reducing therapies, the risk of recurrence remains substantial. Studies, including the POETIC trial, have demonstrated that low Ki67 levels following short-term neoadjuvant endocrine therapy (sNAET) are associated with a favorable prognosis. The objective of this study is to identify genes associated with the suppression of cell cycle progression by sNAET in postmenopausal patients with ER-positive/human epidermal growth factor receptor 2-negative breast cancer.

**Methods:**

Ninety-seven tissue samples were collected and classified into groups based on Ki67 expression levels before and after treatment. RNA sequencing and real-time quantitative reverse transcription PCR were performed to analyze gene expression in tumor samples from patients stratified into High–High (H–H) or High–Low (H–L) groups based on Ki67 levels before and after sNAET.

**Results:**

Among the differentially expressed genes identified, *CXCL9* and *ABCA12* were significantly upregulated in the H–H group and were associated with a poor response to endocrine therapy. Conversely, *NPY1R* was significantly upregulated in the H–L group, suggesting greater responsiveness. In multivariate logistic regression analysis, *CXCL9* (OR: 0.65, *p* = 0.024) and *NPY1R* (OR: 1.61, *p* = 0.048) were significant predictors of Ki67 reduction.

**Conclusion:**

These findings suggest that *CXCL9* and *NPY1R* could serve as predictive biomarkers for endocrine therapy response. Identifying these biomarkers may facilitate personalized treatment strategies, including the addition of therapies such as chemotherapy for resistant cases.

**Supplementary Information:**

The online version contains supplementary material available at 10.1007/s10549-025-07693-8.

## Introduction

Estrogen receptor (ER)-positive breast cancer is the most common clinical subtype, accounting for approximately 80% of cases [1]. In ER-positive breast cancer, postoperative endocrine therapy is the standard treatment, reducing the relative risk of recurrence by 40–50% and breast cancer mortality by 30–40%. In postmenopausal women, aromatase inhibitors (AIs) are the most commonly used agents [2, 3]. However, despite these risk-reducing strategies, the recurrence risk in early-stage ER-positive breast cancer persists for at least 15 years after completing five years of endocrine therapy [4]. Identifying patients who require additional chemotherapy, cyclin-dependent kinase 4/6 inhibitors or extended endocrine therapy remains a critical challenge.

Several multigene assays, including Oncotype DX [5, 6], MammaPrint [7], and EndoPredict [8], provide more accurate prognostic predictions compared to conventional clinicopathological assessments. Additionally, assays such as Oncotype DX [5, 6] can assess the potential benefit of adding chemotherapy to postoperative endocrine therapy. However, studies specifically investigating gene expression profiles predictive of endocrine therapy response remain limited.

Post-treatment Ki67 level is a potential indicator of therapeutic response and prognosis. Studies have shown that if Ki67 levels are adequately suppressed (below 2.7%) following preoperative endocrine therapy, long-term survival can be expected with subsequent postoperative endocrine therapy [9]. Furthermore, the Peri-Operative Endocrine Therapy—Individualising Care (POETIC) trial demonstrated that patients with Ki67 levels below 10% after two weeks of preoperative endocrine therapy had significantly better prognoses than those with levels of 10% or higher [10]. These findings suggest that post-treatment Ki67 levels can help identify patients who could potentially avoid postoperative chemotherapy. On the other hand, when selecting treatment based on Ki67 levels after sNAET, administering sNAET to patients who are inherently unresponsive to endocrine therapy may not only be ineffective but could also potentially be harmful.

Based on these observations, we designed this study to identify genes associated with Ki67 reduction following sNAET using pre-treatment breast tumor tissues from ER-positive/ human epidermal growth factor receptor 2 (HER2)-negative patients.

## Materials and methods

### Study design and population

Ninety-seven tissue samples were collected from postmenopausal women diagnosed with primary ER-positive/ HER2-negative patients invasive breast cancer, with no evidence of distant metastasis. These patients were treated at Kumamoto University Hospital between 2013 and 2023. sNAET lasting 2–8 weeks was recommended while awaiting surgery, based on patients' preferences regarding chemotherapy and lifestyle considerations. The study protocol was approved by the Ethics Committee of the Kumamoto University Graduate School of Medicine (Kumamoto, Japan). All patients underwent tissue biopsies before treatment and received sNAET with one of the following non-steroidal AIs: anastrozole (1 mg daily) or letrozole (2.5 mg daily). Pre-treatment biopsy samples were preserved as formalin-fixed paraffin-embedded (FFPE) tissue blocks, and some were snap-frozen in liquid nitrogen and stored at − 80 °C for subsequent total RNA extraction. Following sNAET, all patients underwent surgery, and the corresponding tumor samples were preserved as FFPE tissue blocks.

**Fig. 1:**
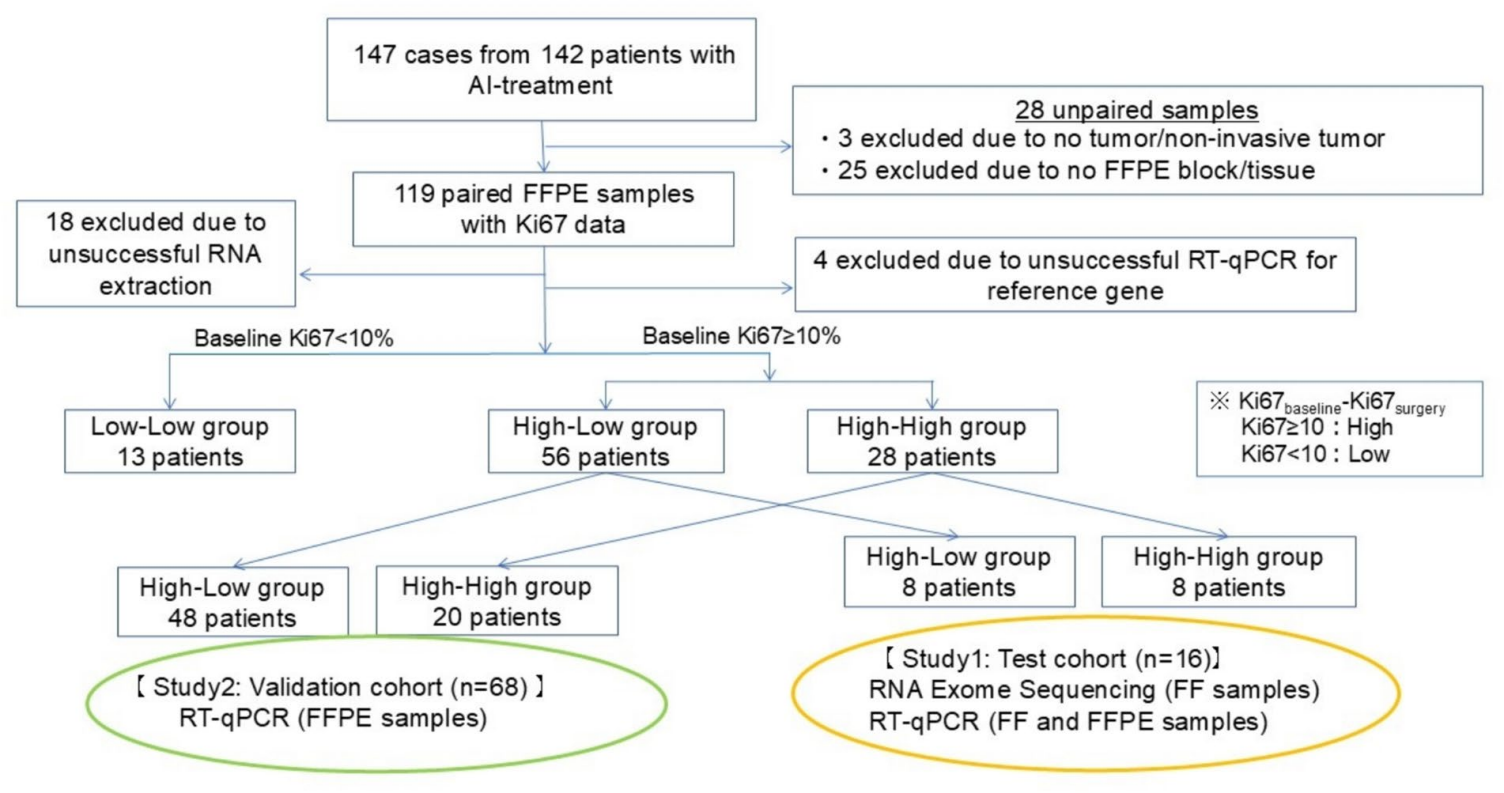
Sample selection process for Study 1 and Study 2. Flowchart showing the selection process of samples from postmenopausal ER + HER2- breast cancer patients treated with AI. *AI* aromatase inhibitor, *ER* + *HER2-* estrogen receptor-positive/human epidermal growth factor receptor 2-negative, *FFPE* formalin-fixed paraffin-embedded

In this study, as shown in Fig. 1, Ki67 values were categorized as High (≥ 10%) or Low (< 10%) at both baseline and surgery. Tumors were classified into three categories based on changes in Ki67 between these time points, as previously reported by Smith et al.: High_baseline_–High_surgery_ (H–H), High_baseline_–Low_surgery_ (H–L), and Low_baseline_–Low_surgery_ (L–L) [10]. To accurately assess the effectiveness of endocrine therapy, our analysis focused on patients with high baseline Ki67 levels (≥ 10%). The L–L group was excluded, as treatment effects were expected to be significant but difficult to distinguish due to consistently low Ki67 levels before and after therapy.

In Study 1, we investigated differences in gene expression between the H–H and H–L groups (eight patients per group) following sNAET using RNA sequencing (RNA-seq) on fresh frozen (FF) tissue samples. When significant differences in gene expression were identified through RNA-seq, real-time quantitative reverse transcription PCR (RT-qPCR) was performed on both FF and FFPE tissue samples from the same cases to validate the correlation and significance of the DEGs.

In Study 2, we further validated the DEGs identified in Study 1 by performing RT-qPCR on FFPE tissue samples from an independent validation cohort, consisting of 48 patients in the H–H group and 20 in the H–L group.

### Immunohistochemical analysis

Immunohistochemical (IHC) staining was performed on FFPE tumor tissue samples to quantitatively assess the expression levels of ER, progesterone receptor (PgR), HER2, and Ki67. The following monoclonal antibodies were used: ERα (SP1; Ventana Japan, Tokyo, Japan), PgR (1E2; Ventana Japan), HER2 (4B5; Roche, Tokyo, Japan), and Ki67 (MIB1; Dako Japan, Kyoto, Japan). Staining was conducted using the NexES IHC immunostainer (Ventana Medical Systems, Tucson, AZ, USA) according to the manufacturer’s protocols. A sample was considered positive for ER and PgR if more than 1% of tumor cell nuclei were stained. HER2 expression was assessed using the HercepTest, with tumors classified as HER2-positive if they exhibited a 3 + score by IHC or demonstrated a greater than 2.0-fold increase in fluorescence in situ hybridization (FISH) or dual-color in situ hybridization (DISH). Ki67 expression was quantified as the percentage of stained nuclei among all cancer cells at the invasive tumor margin, irrespective of staining intensity, in a × 400 high-power field. Per the recommendations of the International Ki67 in Breast Cancer Working Group [11], 500 to 1,000 tumor cells were counted per sample.

### RNA extraction and assessment of quality

RNA was extracted from 16 FF specimens snap-frozen in liquid nitrogen using the AllPrep DNA/RNA/miRNA Universal Kit (QIAGEN, Hilden, Germany) according to the manufacturer’s instructions. All tissue samples had been previously fixed in 10% neutral-buffered formalin for a maximum of 72 h. Total RNA was extracted from 4 × 10 μm FFPE tissue sections using the AllPrep DNA/RNA FFPE Kit (QIAGEN), following the manufacturer’s instructions, with tumor areas selectively dissected using a sterilized blade.

The percentage of tumor cells in each FFPE block was assessed on stained tissue sections to confirm the diagnosis and ensure a tumor content of over 50%. Total RNA quantification was performed using a NanoDrop 2000 spectrophotometer (Thermo Fisher Scientific, Waltham, MA, USA) by measuring the A260/A280 absorbance ratios. RNA quality was evaluated using an Agilent 2100 Bioanalyzer (Agilent Technologies, Santa Clara, CA, USA) with an Agilent RNA 6000 Nano Kit (Agilent Technologies). The Agilent RNA integrity number (RIN) values were > 7 for FF tissue and < 3 for FFPE tissue. RNA samples were stored at − 80 °C until further analysis.

### RNA sequencing

RNA-seq was performed on total RNA extracted using the AllPrep DNA/RNA/miRNA Universal Kit (QIAGEN). Sequencing libraries were prepared using the Illumina TruSeq RNA Exome Library Prep Kit (Illumina, San Diego, CA, USA), and RNA-seq was conducted on a NovaSeq 6000 (Illumina) using paired-end sequencing with a read length of 100 base pairs, following the manufacturer’s instructions. Library preparation and sequencing were performed by RIKEN GENESIS Co., Ltd. (Tokyo, Japan), a facility specializing in advanced genomics technologies.

For RNA-seq data analysis, raw sequencing reads were first assessed for quality, detecting potential sequencing errors and contaminants using FastQC (Babraham Institute, Cambridge, UK). Adapter sequences and low-quality bases were trimmed using Cutadapt [12], followed by the removal of polyA tails and polyN sequences using PRINSEQ [13]. Reads shorter than 20 base pairs after trimming were excluded from further analysis. The remaining reads were aligned to the reference genome (iGenomes *Homo sapiens* NCBI/build37.2) using TopHat (version 2.1.1), with annotated genes serving as the transcriptome index. Cufflinks (version 2.2.1) was used to assemble aligned reads into transcripts, quantify their abundance, and identify differences in gene expression across samples [14].

Gene expression levels were calculated in fragments per kilobase of transcript per million mapped reads (FPKM), a metric that normalizes for transcript length and total read count, enabling direct comparison across samples. Differential expression analysis between groups was conducted using Cuffdiff, with significant DEGs identified based on a q-value threshold (false discovery rate (FDR)-adjusted *p*-value) of 0.05. Transcripts with very low abundance (total FPKM < 10 across both groups) were excluded. Additionally, DEGs with minor expression changes were filtered out using a minimum log₂ fold change (FC) threshold of ± 1.5.

### Real-time quantitative reverse transcription PCR

Total RNA was reverse transcribed to cDNA using the PrimeScript® RT Master Mix (Takara Bio, Shiga, Japan) according to the manufacturer's protocol. RT-qPCR was performed on the QuantStudio™ 12 K Flex Real-Time PCR System (Applied Biosystems, Foster City, CA, USA) using TaqMan™ Fast Advanced Master Mix (Applied Biosystems) and Thermo Fisher Assay on Demand primer–probe kits. The assays for target genes selected for this study and the four reference genes are shown in Table S1. Relative mRNA levels were calculated from the threshold cycle for amplification using the ΔΔCt method. Cycle threshold (Ct) values were measured in triplicate and normalized to the Ct values of simultaneous triplicate measurements of the expression of four reference genes (*ACTB*, *PUM1*, *TAF-10*, and *FKBP15*) from the same samples. These reference genes were selected based on our previous study [15].

### Statistical analysis

Pearson’s chi-squared test, Fisher’s exact test, and the nonparametric Wilcoxon rank-sum test were used to compare clinicopathological variables and assess differences in variable distributions among groups. For RNA-seq data, Student’s *t*-test was used to identify significant differences in FC values between groups, with significance defined as an FC of ± 1.5 or greater and an FDR-adjusted *p*-value < 0.05.

Spearman’s rank correlation coefficients were calculated to assess the correlation of mRNA expression levels between FFPE and FF specimens. For RT-qPCR analysis, the Wilcoxon rank-sum test was applied to compare gene expression levels between the H–H and H–L groups.

To predict a decrease in Ki67 levels (< 10%) following sNAET, logistic regression analysis was performed to evaluate the association between gene expression levels and Ki67 reduction. Odds ratios (ORs) and 95% confidence intervals (CIs) were calculated.

All statistical analyses were performed using JMP Pro version 17 (SAS Institute Inc., Cary, NC, USA). All tests were two-sided, and *p*-values < 0.05 were considered statistically significant.

## Results

### Patients and tumor characteristics

Of the 147 total cases, 97 FFPE specimens (66%) had detectable levels of all four reference genes, with Ct values < 40, and were selected for this study. The remaining 50 cases were excluded due to the inability to evaluate Ki67 levels in either baseline or surgical samples (n = 28), unsuccessful RNA extraction (n = 18), or abnormal Ct values for one or two reference genes (n = 4) (Fig. 1).

In Study 1, the median treatment duration was 22 days (range: 10–40 days), whereas in Study 2, it was 27 days (range: 10–52 days). The baseline characteristics of the patients in Study 1 and Study 2 are summarized in Table 1. In Study 1, confounding factors were minimized as much as possible to enable a direct comparison of gene expression between the H–H and H–L groups, resulting in no significant clinicopathological differences between the two groups.

**Table 1:** Clinical and pathological characteristics of patients treated with sNAET in Study 1 and Study2

Characteristics	Study1			Study2		
	H–L (n = 8)	H–H (n = 8)	*p*-value	H–L (n = 48)	H–H (n = 20)	*p*-value
Age at diagnosis, median (range)	68 (54–81)	59 (53–75)	0.17	69 (51–85)	69 (52–81)	0.80
Treatment duration (days), median (range)	20 (10–40)	22 (14–37)	0.92	28 (10–50)	23 (10–52)	0.47
BMI, n (%)			0.28			0.74
< 22	4 (50)	1 (12.5)		8 (16.7)	4 (20)	
≥ 22	4 (50)	7 (87.5)		40 (83.3)	16 (80)	
Tumor size (mm), n (%)			1.00			0.16
≤ 20	2 (25)	3 (37.5)		34 (70.8)	10 (50)	
> 20	6 (75)	5 (62.5)		14 (29.2)	10 (50)	
Nodal status, n (%)			0.47			1.00
negative	6 (75)	8 (100)		44 (91.7)	18 (90)	
positive	2 (25)	0 (0)		4 (8.3)	2 (10)	
Histological type, n (%)			1.00			0.78
IDC	7 (87.5)	8 (100)		45 (93.8)	18 (90)	
ILC	0 (0)	0 (0)		2 (4.2)	1 (5)	
others	1 (12.5)	0 (0)		1 (2.1)	1 (5)	
ER _baseline_ (%), median (IQR)	95 (91, 99)	95 (90, 99)	0.66	100 (95, 100)	95 (91, 100)	0.051
PgR _baseline_ (%), median (IQR)	70 (12.5, 77.5)	20 (0, 55)	0.15	90 (52.5, 95)	77.5 (15, 90)	0.17
Ki67 _baseline_ (%), median (IQR)	18 (14, 26)	22 (16, 42)	0.19	20 (13, 24)	32 (22, 42)	< 0.001
Histological grade, n (%)			0.57			0.014
I	2 (25)	0 (0)		14 (29.2)	1 (5)	
II	5 (62.5)	6 (75)		29 (60.4)	12 (60)	
III	1 (12.5)	2 (25)		5 (10.4)	7 (35)	
Stage, n (%)			1.00			0.068
I	1 (12.5)	2 (25)		34 (70.8)	9 (45)	
II	7 (87.5)	6 (75)		11 (22.9)	10 (50)	
III	0 (0)	0 (0)		3 (6.3)	1 (5)	

*BMI* Body mass index, *ER* Estrogen receptor, *H–H* High Ki67_baseline_—High Ki67_surgery_, *H–L* High Ki67_baseline_—Low Ki67_surgery_, *IDC* Invasive ductal carcinoma, *ILC* Invasive lobular carcinoma, *IQR* Interquartile range, *sNAET* Short-*term* neoadjuvant endocrine therapy, *PgR*, Progesterone receptor

In contrast, Study 2 involved a larger validation cohort of 68 patients, allowing for a more comprehensive evaluation of clinical factors to validate the findings from Study 1. Similar to Study 1, efforts were made to adjust for confounding factors; however, unlike Study 1, significant differences were observed between the H–H and H–L groups in terms of Ki67 levels (*p* < 0.001) and histological grade (*p* = 0.014).

### Identification of differentially expressed genes by RNA-seq

We conducted a comprehensive gene expression analysis and identified several DEGs between the H–H and H–L groups prior to sNAET. Table 2 presents the DEGs among the 14,319 genes analyzed by RNA-seq (see Supplementary Fig. 1). In the H–H group, five genes (*CDH2, CDSN, ABCA12, CLGN,* and *CXCL9*) were upregulated, while eight genes (*SLC18A2, PCDH19, NPY1R, ANPEP, CXCL14, DUSP4, STEAP4,* and *THBS4*) were downregulated. Non-coding RNAs, including microRNAs (miRNAs) and small nucleolar RNAs (SNORDs), as well as genes with very low FPKM values, were excluded from further analysis, particularly for RT-qPCR validation using FFPE samples. These non-coding RNAs were unsuitable for RT-qPCR validation due to their low expression levels, high RNA degradation rates in FFPE samples, and technical challenges associated with their short length, which negatively impact reverse transcription efficiency and complicate primer design for accurate quantification.

**Table 2:** Differentially expressed genes related to patients in the H–H group compared with the H–L group, as determined by RNA sequencing

Gene name	Log2 (fold change)	*q-values *^a^ *(FDR-adjusted p-values)*
Upregulated genes		
*CDH2*	5.41	0.018
*CDSN*	3.84	0.018
*ABCA12*	2.88	0.046
*CLGN*	2.66	0.018
*CXCL9*	1.90	0.039
Downregulated genes		
*SLC18A2*	− 5.64	0.018
*PCDH19*	− 5.59	0.018
*NPY1R*	− 4.99	0.018
*ANPEP*	− 2.86	0.018
*CXCL14*	− 2.38	0.032
*DUSP4*	− 2.28	0.018
*STEAP4*	− 2.14	0.029
*THBS4*	− 2.01	0.032

*FDR* False discovery rate, *H–H* High Ki67_baseline_—High Ki67_surgery,_ *H–L* High Ki67_baseline_—Low Ki67_surgery_

^a^Only genes with a statistically significant q-value (q < 0.05) are shown

### Validation of differential gene expression in FF and FFPE samples by RT-qPCR

To validate the DEGs identified through RNA-seq, RT-qPCR was performed on both FF and FFPE samples obtained from the same cases in Study 1. A significant positive correlation was observed between gene expression levels in FF and FFPE samples for most DEGs, with Spearman’s correlation coefficients (ρ) ranging from 0.50 to 0.94, indicating a high degree of consistency between the two tissue preservation methods (Supplementary Fig. 2). The strongest correlations were observed for *THBS4* (ρ = 0.94, *p* < 0.001), *CXCL9* (ρ = 0.93, *p* < 0.001), *CLGN* (ρ = 0.90, *p* < 0.001), *ABCA12* (ρ = 0.89, *p* < 0.001), and *ANPEP* (ρ = 0.89, *p* < 0.001). Relatively strong correlations were also noted for *DUSP4* (ρ = 0.78, *p* < 0.001), *CDH2* (ρ = 0.78, *p* < 0.001), *NPY1R* (ρ = 0.70, *p* < 0.01), *CXCL14* (ρ = 0.62, *p* < 0.01), *STEAP4* (ρ = 0.58, *p* = 0.019), and *SLC18A2* (ρ = 0.50, *p* = 0.048). However, the presence of outliers affected the correlation and statistical significance for certain genes, such as *PCDH19* and *CDSN*, reducing the correlation to ρ = 0.44 (*p* = 0.091) for *PCDH19* and ρ = 0.51 (*p* = 0.16) for *CDSN*.

### Validation of DEGs using FFPE in the validation cohort

**Fig. 2:**
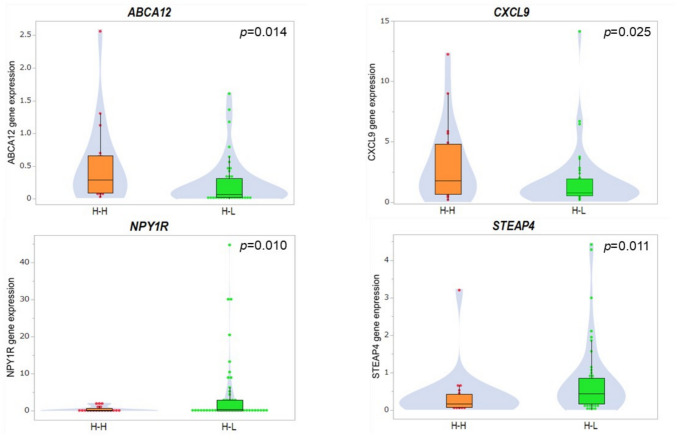
Validation of differential gene expression between the High-High and High-Low groups in response to sNAET, as determined by RT-qPCR. Only genes with statistically significant differences in expression (*p* < 0.05) are shown. Gene expression levels were analyzed using the Wilcoxon rank-sum test. *H–H* High Ki67_baseline_—High Ki67_surgery_, *H–L* High Ki67_baseline_—Low Ki67_surgery_, *sNAET* short-term neoadjuvant endocrine therapy

The DEGs identified through RNA-seq were further validated using RT-qPCR in a separate validation cohort (Study 2), as shown in Fig. 2. *ABCA*12 and *CXCL9* showed significantly higher expression in the H–H group compared to the H–L group (p = 0.014 and p = 0.025, respectively). In contrast, *NPY1R* and *STEAP4* exhibited significantly higher expression in the H–L group than in the H–H group (*p* = 0.010 and *p* = 0.011, respectively). No statistically significant differences were observed in the expression levels of the other genes between the H–H and H–L groups (Supplementary Fig. 3).

Logistic regression analysis was performed to evaluate the predictive value of mRNA expression levels for a decrease in Ki67 levels (< 10%) following sNAET. In the univariate analysis, *ABCA12* (OR: 0.20, 95% CI: 0.06–0.62, *p* = 0.006) and *CXCL9* (OR: 0.68, 95% CI: 0.53–0.88, *p* = 0.003) were negatively associated with Ki67 reduction, while *NPY1R* (OR: 1.81, 95% CI: 1.03–3.18, *p* = 0.039) showed a positive association. *STEAP4* (OR: 2.79, 95% CI: 0.95–8.17, *p* = 0.062) showed a trend but did not reach statistical significance. In multivariate analysis, only *CXCL9* (OR: 0.65, 95% CI: 0.44–0.94, *p* = 0.024) and *NPY1R* (OR: 1.61, 95% CI: 1.00–2.59, *p* = 0.048) remained significant predictors (Table 3).

**Table 3:** Logistic regression analysis of mRNA expression by RT-qPCR for predicting a decrease in Ki67 levels (all cohort, n = 84)

	Univariate analysis			Multivariate analysis	
	OR (95% CI)		*p*-value^a^	OR (95% CI)	*p*-value^a^
*ABCA12*		0.20 (0.06–0.62)	0.006	0.44 (0.11–1.70)	0.23
*CXCL9*		0.68 (0.53–0.88)	0.003	0.65 (0.44–0.94)	0.024
*NPY1R*		1.81 (1.03–3.18)	0.039	1.61 (1.00–2.59)	0.048
*STEAP4*		2.79 (0.95–8.17)	0.062	1.67 (0.51–5.47)	0.40

*CI* Confidence interval, *OR* Odds ratio

^a^*p*-value < 0.05 indicates statistical significance

Additionally, in multivariate analysis (Table 4), after adjusting for baseline Ki67 levels, tumor stage, and treatment duration, *CXCL9* (OR: 0.64, 95% CI: 0.41–0.89, p = 0.030) and *NPY1R* (OR: 1.52, 95% CI: 1.09–3.01, p = 0.047) remained significant independent predictors of Ki67 reduction. In contrast, baseline Ki67 levels, tumor stage, and treatment duration were not independently predictive of Ki67 reduction (p > 0.05).

**Table 4:** Multivariate logistic regression analysis of clinicopathological factors influencing a decrease in Ki67 levels (all cohort, n=84)

	OR (95% CI)	*p*-value^a^
*CXCL9*	0.64 (0.41–0.89)	0.030
*NPY1R*	1.52 (1.09–3.01)	0.047
Baseline Ki67 level	0.94 (0.87–1.01)	0.062
Stage (I vs. II–III)	2.08 (0.61–7.44)	0.24
Treatment duration of sNAET (≥ 4 vs. < 4 weeks)	1.65 (0.46–6.33)	0.44

*CI* Confidence interval, *OR* Odds ratio

^a^*p*-value < 0.05 indicates statistical significance

## Discussion

In this study, we identified DEGs associated with Ki67 reduction following sNAET in postmenopausal women with ER-positive/HER2-negative breast cancer. Previous studies have primarily focused on Ki67 as a prognostic marker after neoadjuvant endocrine therapy; however, specific genes predictive of Ki67 reduction have not been well established. This study provides new insights by identifying *CXCL9* and *NPY1R* as potential surrogate markers for predicting Ki67 reduction following sNAET.

*CXCL9* was significantly upregulated in the H–H group, where Ki67 levels remained high after sNAET, suggesting that *CXCL9* may be associated with poor reduction in Ki67 levels. *CXCL9*, a chemokine involved in modulating immune responses by promoting tumor-infiltrating lymphocytes (TILs), has been associated with a favorable prognosis in ER-negative/HER2-negative breast cancer, correlating with improved survival and higher TIL levels [16, 17]. However, recent studies indicate that in ER-positive/HER2-negative breast cancer, higher *CXCL9* expression is associated with a poorer prognosis [18, 19]. In our study, elevated *CXCL9* expression was linked to higher Ki67 levels in the H–H group.

In contrast, *NPY1R* was upregulated in the H–L group, where Ki67 levels decreased after sNAET, suggesting that *NPY1R* may serve as a predictive marker for Ki67 reduction following endocrine therapy. *NPY1R* has been associated with improved relapse-free and overall survival in ER-positive breast cancer, with elevated expression in estrogen-sensitive subtypes such as Luminal A. Bhat et al. reported that NPY treatment suppressed estradiol-stimulated cell growth, and this effect was reversed by an *NPY1R* antagonist, supporting the role of *NPY1R* in modulating endocrine sensitivity [20].

However, several limitations must be considered. The relatively small sample size, variability in treatment duration across cases, and the retrospective nature of this study are primary limitations that may affect the generalizability and robustness of our findings. The small sample size of the RNA-seq cohort (n = 16) is a major limitation, which increases the risk of overfitting and reduces the statistical power of the analysis. To minimize overfitting, RNA-seq results were validated by RT-qPCR in an independent cohort (n = 68). Nonetheless, larger sample sizes and external validation are needed to strengthen the generalizability of these findings.

We also acknowledge that the retrospective nature of the study introduces potential biases, including selection bias and confounding factors. Multivariate logistic regression was used to adjust for key confounders, including tumor stage, baseline Ki67 levels, and treatment duration. Even after adjusting for these confounders, *CXCL9* and *NPY1R* remained independent predictors of Ki67 reduction following sNAET, reinforcing the validity of these findings. However, the absence of a well-matched control group remains a significant limitation. Furthermore, the variability in treatment duration across cases may reflect insufficient adjustment for the waiting period before surgery.

Also, tissue preservation methods pose a significant challenge. RNA degradation in FFPE samples may have affected the accuracy of gene expression measurements, particularly for low-expressed genes. This issue was especially pronounced for non-coding RNAs, such as miRNAs and SNORDs, due to their low expression levels, short length, and increased susceptibility to degradation in FFPE samples [21–23]. These factors collectively complicate the detection and quantification of these RNAs, ultimately impacting the reliability of RT-qPCR results.

Another limitation is that mRNA expression does not always reflect protein levels. Our analysis focused exclusively on gene expression at the mRNA level. While mRNA data provide valuable insights into transcriptional changes, they do not always correlate with protein-level activity, which is often more directly linked to biological function. Therefore, future studies should include IHC to evaluate protein expression levels of *CXCL9* and *NPY1R* in a larger, independent cohort to confirm their clinical relevance.

Furthermore, the biological mechanisms by which *CXCL9* and *NPY1R* regulate Ki67 reduction remain unclear. In vitro and in vivo experiments are needed to clarify whether *CXCL9* and *NPY1R* directly influence Ki67 expression and cell proliferation. For example, manipulating *NPY1R* expression levels (overexpression or knockdown) in ER-positive breast cancer cell lines could help determine the downstream signaling pathways involved in Ki67 regulation. Similarly, targeting *CXCL9* or its signaling pathway in mouse models may provide insights into its role in endocrine therapy response. These functional studies will help to establish whether *CXCL9* and *NPY1R* are predictive biomarkers for Ki67 reduction.

Additionally, the number of recurrence events in this cohort was too small to evaluate the correlation of *CXCL9* and *NPY1R* expression with progression-free survival (PFS). However, analysis using the Kaplan–Meier plotter (https://kmplot.com/analysis/) showed that high *NPY1R* expression was significantly associated with longer PFS, while high *CXCL9* expression was significantly associated with shorter PFS in ER-positive breast cancer (Supplementary Fig. 4).

If validated in larger cohorts, these findings could facilitate more tailored treatment strategies by identifying patients unlikely to benefit from endocrine therapy alone, thereby guiding the need for additional therapies such as chemotherapy or targeted therapies.

In conclusion, this study identified *CXCL9* and *NPY1R* as potential surrogate markers for predicting Ki67 reduction following sNAET in ER-positive/HER2-negative breast cancer. *CXCL9* was associated with poor Ki67 reduction, whereas *NPY1R* was linked to improved Ki67 response. These findings suggest that *CXCL9* and *NPY1R* may serve as predictive biomarkers for endocrine therapy response. However, the small sample size, retrospective design, and lack of external validation are key limitations that require further investigation.

Future studies with larger, prospective, multi-center cohorts and functional experiments are necessary to validate these findings and confirm their utility as predictive markers of Ki67 reduction. Moreover, elucidating the biological roles of *CXCL9* and *NPY1R* at the protein level through IHC and functional assays will be essential for clinical translation.

## Supplementary Information

Below is the link to the electronic supplementary material.

Supplementary file1 (PDF 545 KB)

## Data Availability

The datasets used in this study are available from the corresponding author upon reasonable request.

## Abbreviations

ABCA12: ATP binding cassette subfamily A member 12
ANPEP: Alanyl aminopeptidase, membrane
CDH2: Cadherin 2
CDSN: Corneodesmosin
CLGN: Calmegin
CXCL9: C-X-C motif chemokine ligand 9
CXCL14: C-X-C motif chemokine ligand 14
DEG: Differentially expressed gene
DUSP4: Dual specificity phosphatase 4
NPY1R: Neuropeptide Y receptor Y1
PCDH19: Protocadherin 19
SLC18A2: Solute carrier family 18 member A2
STEAP4: Six-transmembrane epithelial antigens of prostate
sNAET: Short-term neoadjuvant endocrine therapy
THBS4: Thrombospondin 4
