# Supplementary material for: Identifying gene expression predictive of response to neoadjuvant endocrine therapy in early breast cancer
Source: Breast Cancer Res Treat. 2025 Apr 9;211(3):717–25. doi: 10.1007/s10549-025-07693-8 (PMC12031974; doi:10.1007/s10549-025-07693-8)
Supplement: Supplementary file 1 — Supplementary file1 (PDF 545 KB) [file 10549_2025_7693_MOESM1_ESM.pdf]

Identifying gene expression predictive of response to neoadjuvant endocrine therapy in early breast cancer

Breast Cancer Research and Treatment

Kaori Hidaka[1,2], Lisa Goto-Yamaguchi[2], Aiko Sueta[3], Mai Tomiguchi[1], Yutaka Yamamoto[2]

[1]Department of Thoracic Surgery and Breast Surgery, Graduate School of Medical Sciences, Kumamoto University, Kumamoto, Japan
[2]Department of Breast and Endocrine Surgery, Kumamoto University Hospital, Kumamoto, Japan
[3]Department of Breast Surgery, Wajiro Hospital, Fukuoka, Japan

Corresponding author:
Yutaka Yamamoto
E-mail: yyamamoto@kumamoto-u.ac.jp

Supplementary Figure 1 Volcano plot of differentially expressed genes prior to short-term aromatase inhibitor (AI) treatment. This volcano plot shows the results of the RNA sequencing analysis of 14,319 genes comparing gene expression between the H-H and H-L groups before AI treatment. The x-axis represents the Log2 fold change in gene expression, while the y-axis shows the -Log10(*p*-value). Genes significantly upregulated in the H-H group are shown on the right side of the plot (e.g., *CDH2*, *CDSN*, *ABCA12*, *CLGN*, and *CXCL9*), while downregulated genes in the H-H group are displayed on the left side (e.g., *SLC18A2*, *PCDH19*, *NPY1R*, *ANPEP*, *CXCL14*, *DUSP4*, *STEAP4*, and *THBS4*).

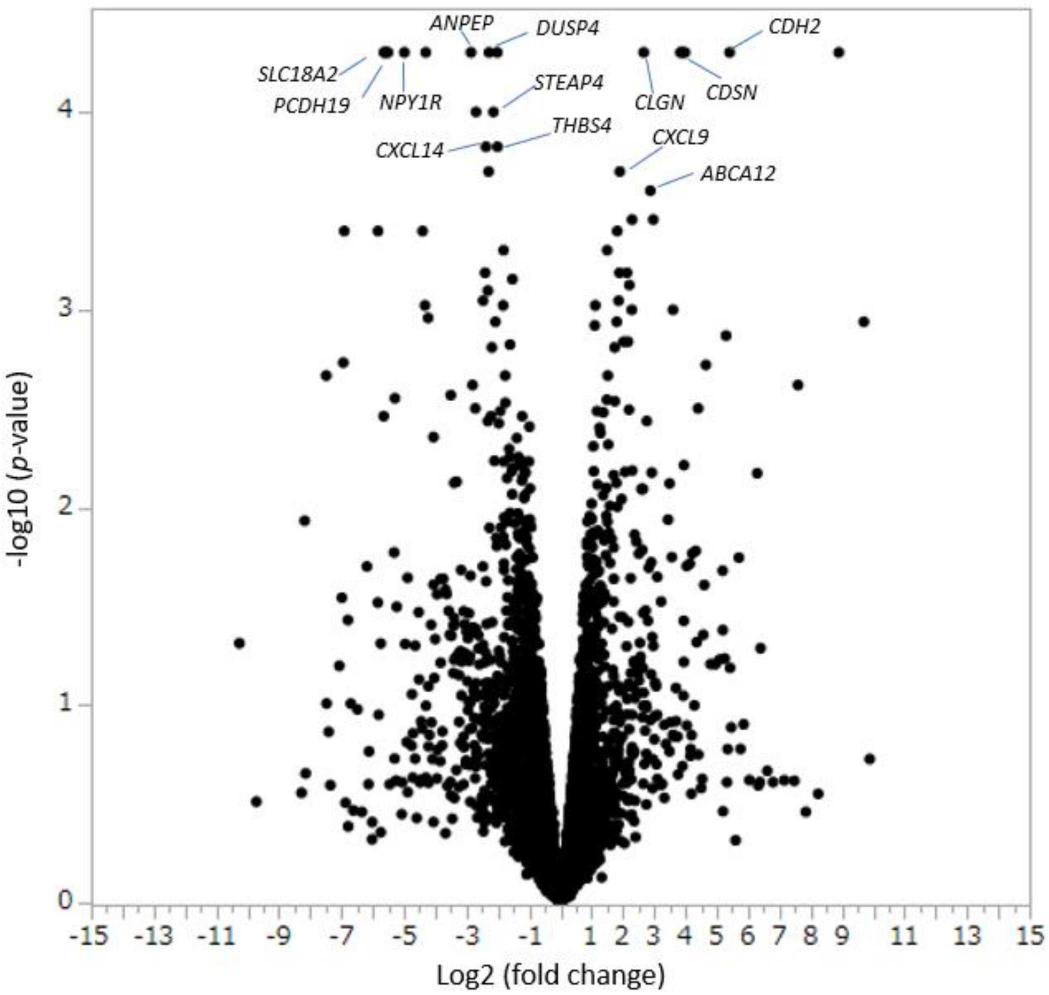

Supplementary Figure 2 Correlation between FFPE and FF for the relative quantification of DEGs using RT-qPCR. This figure shows the correlation of gene expression levels between FFPE and FF samples for each DEG identified by RNA-seq in Study 1. *DEG* differentially expressed gene, *FF* fresh frozen, FFPE formalin-fixed paraffin-embedded

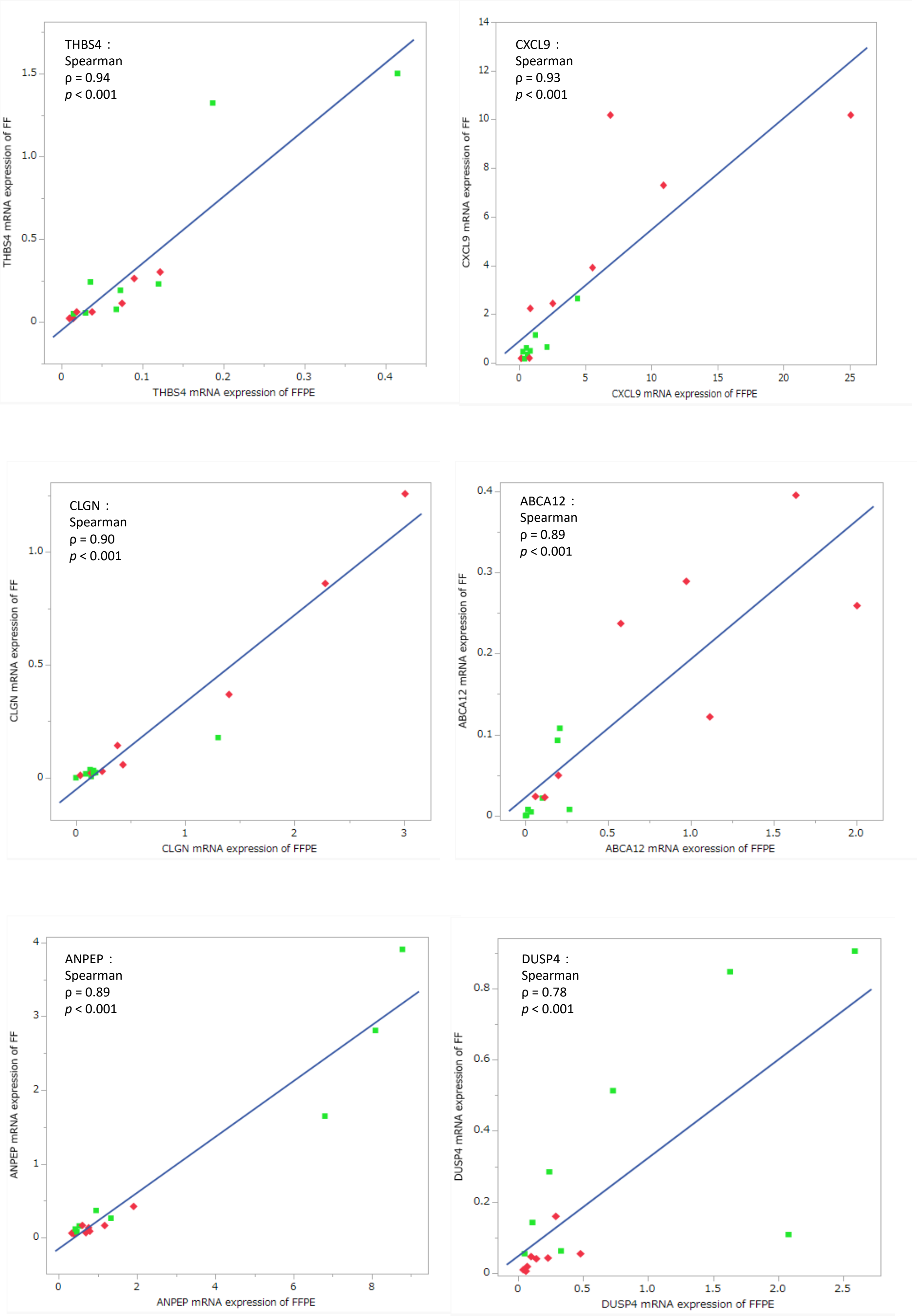

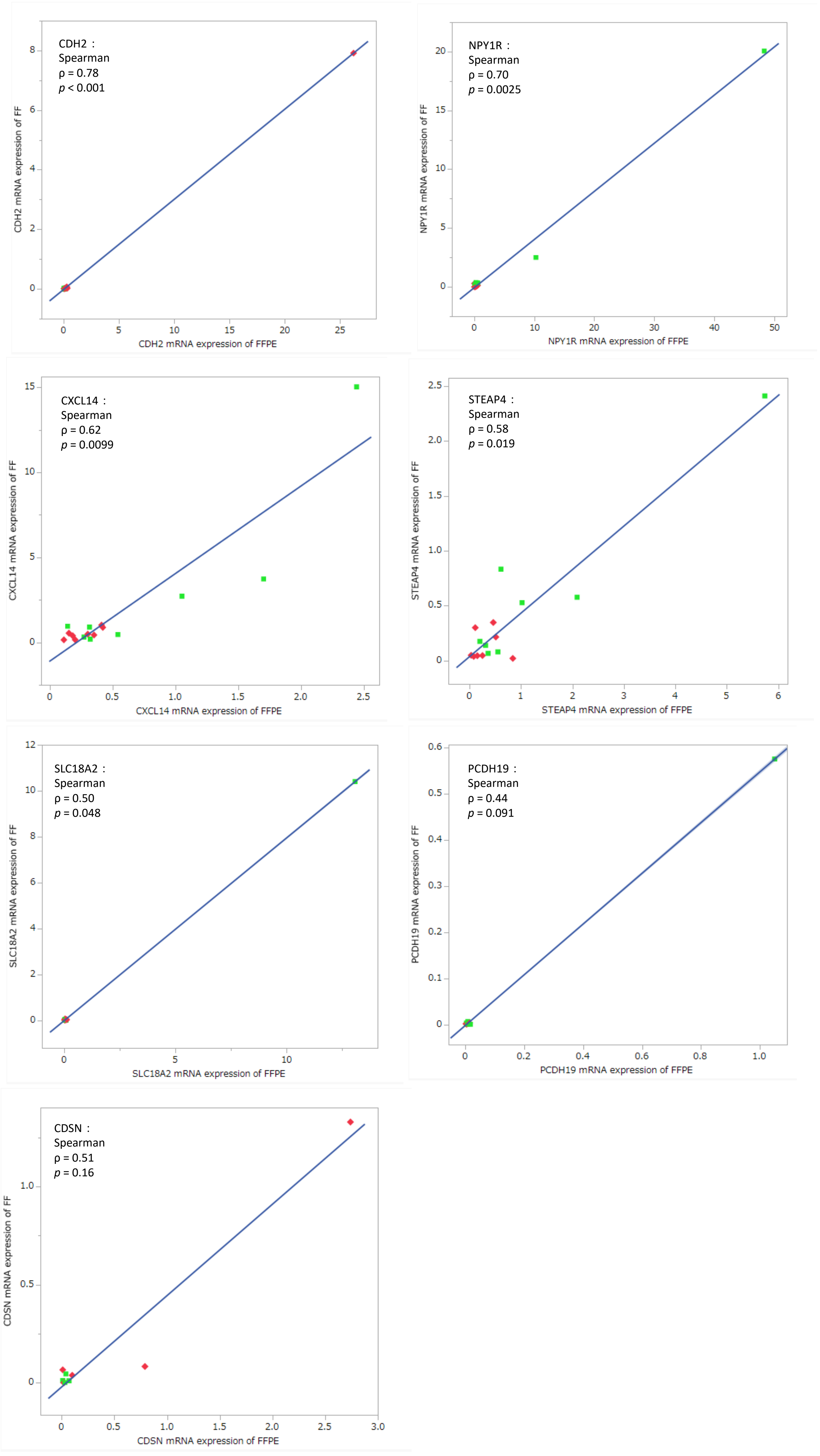

CDH2 :
Spearman
ρ = 0.78
p < 0.001
CDH2 mRNA expression of FF
CDH2 mRNA expression of FFPE
NPY1R :
Spearman
ρ = 0.70
p = 0.0025
NPY1R mRNA expression of FF
NPY1R mRNA expression of FFPE
CXCL14 :
Spearman
ρ = 0.62
p = 0.0099
CXCL14 mRNA expression of FF
CXCL14 mRNA expression of FFPE
STEAP4 :
Spearman
ρ = 0.58
p = 0.019
STEAP4 mRNA expression of FF
STEAP4 mRNA expression of FFPE
SLC18A2 :
Spearman
ρ = 0.50
p = 0.048
SLC18A2 mRNA expression of FF
SLC18A2 mRNA expression of FFPE
PCDH19 :
Spearman
ρ = 0.44
p = 0.091
PCDH19 mRNA expression of FF
PCDH19 mRNA expression of FFPE
CDSN :
Spearman
ρ = 0.51
p = 0.16
CDSN mRNA expression of FF
CDSN mRNA expression of FFPE

Supplementary Figure 3 Validation of gene expression differences between the H-H and H-L groups in response to short-term neoadjuvant endocrine therapy, as determined by RT-qPCR in Study 2. The expression levels of nine genes identified as differentially expressed genes via RNA sequencing were analyzed. No statistically significant differences were observed in the expression levels of these genes between the H-H and H-L groups (all *p*-values > 0.05)

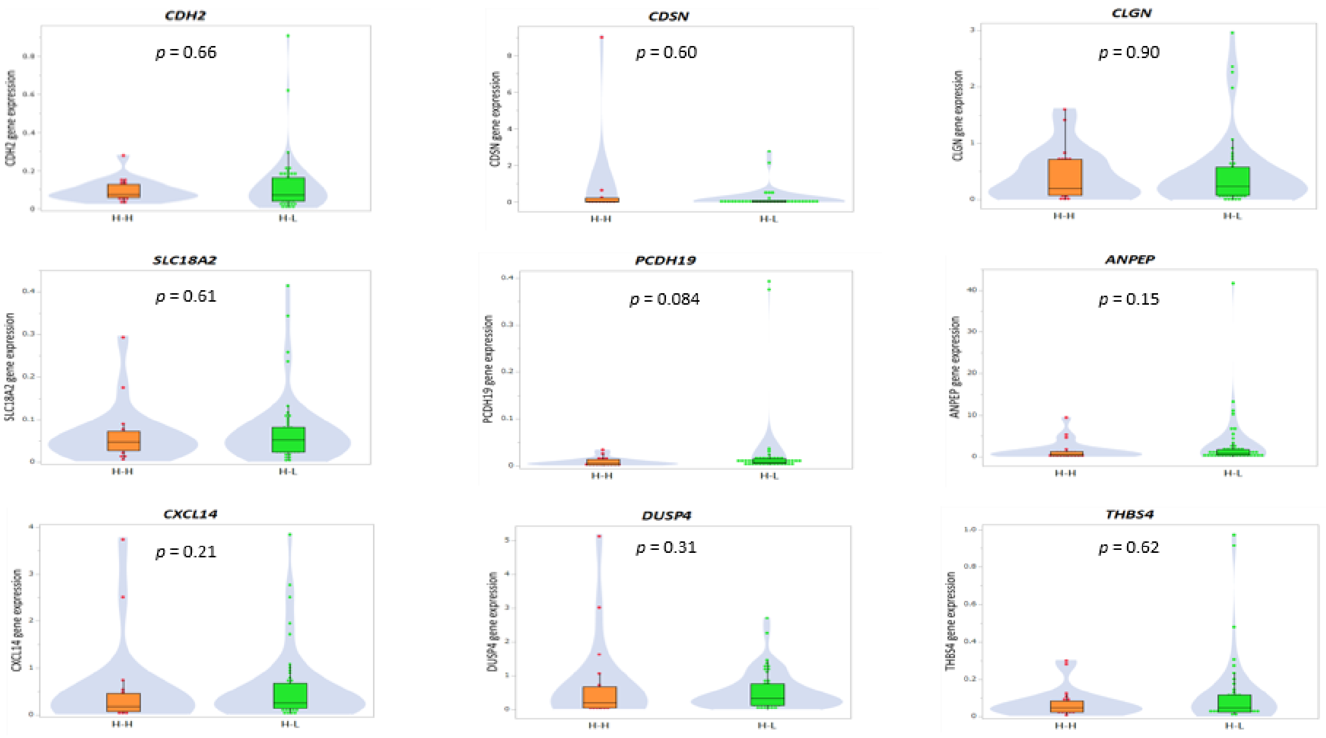

Supplementary Figure 4 Prognostic values of CXCL9 and NPY1R expression. RFS was evaluated using an online database, Kaplan-Meier plotter. The cut-off values are set at the median for each gene. Log-rank p-value < 0.05 was considered statistically significant. Red lines, higher than median gene expression; black line, lower than median gene expression. *HR* hazard ratio

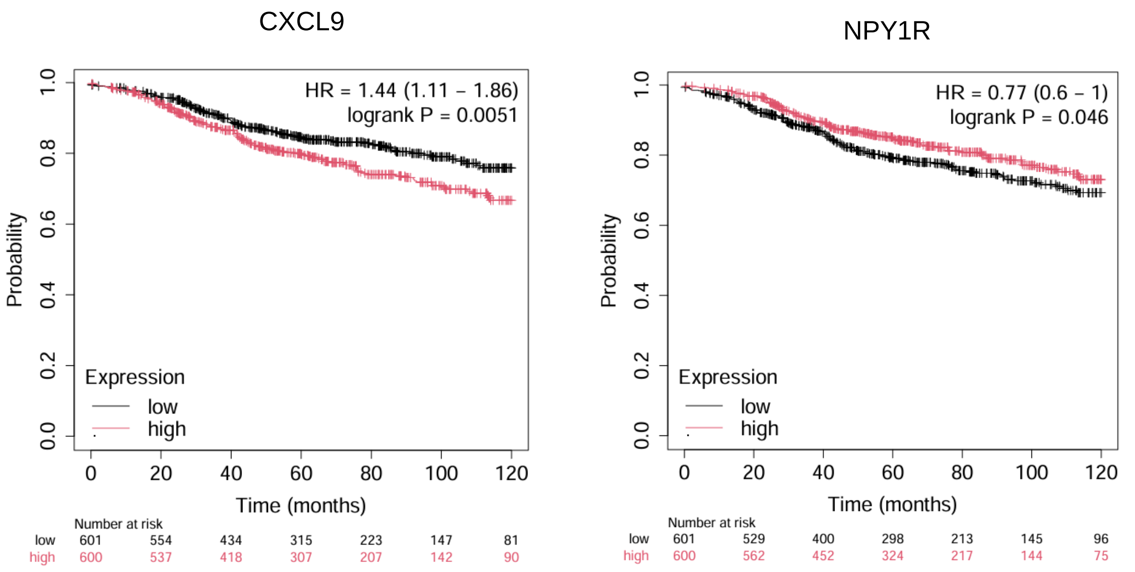

Supplementary Table 1 TaqMan gene expression assay of 13 genes selected for this study and four reference genes

Listing of the 13 selected genes		
Gene	Gene name	TaqMan assay ID
CDH2	cadherin 2	Hs00983056_m1
CDSN	corneodesmosin	Hs00169911_m1
ABCA12	ATP binding cassette subfamily A member 12	Hs00292421_m1
CLGN	calmegin	Hs00189073_m1
CXCL9	C-X-C motif chemokine ligand 9	Hs00171065_m1
SLC18A2	solute carrier family 18 member A2	Hs00996844_m1
PCDH19	protocadherin 19	Hs00943207_m1
NPY1R	neuropeptide Y receptor Y1	Hs00702150_s1
ANPEP	alanyl aminopeptidase, membrane	Hs00174265_m1
CXCL14	C-X-C motif chemokine ligand 14	Hs01557413_m1
DUSP4	dual specificity phosphatase 4	Hs01027785_m1
STEAP4	six-transmembrane epithelial antigens of prostate	Hs01026584_m1
THBS4	thrombospondin 4	Hs00170261_m1
Listing of the four reference genes		
ACTB	actin beta	Hs01060665_g1
FKBP15	FK506 binding protein 15	Hs00910471_m1
TAF-10	TATA-box binding protein associated factor 10	Hs00359540_g1
PUM1	pumilio RNA binding family member 1	Hs00982775_m1
